# Effects of carbon source addition in rearing water on sediment characteristics, growth and health of cultured marron (*Cherax cainii*)

**DOI:** 10.1038/s41598-024-51585-8

**Published:** 2024-01-16

**Authors:** Thi Thu Thuy Nguyen, Md Javed Foysal, Sanjay Kumar Gupta, Alfred Tay, Ravi Fotedar, Marthe Monique Gagnon

**Affiliations:** 1 School of Molecular and Life Sciences, Curtin University, Bentley, WA Australia; 2 Department of Experimental Biology, Research Institute for Aquaculture No.2, Ho Chi Minh City, Vietnam; 3 Department of Genetic Engineering and Biotechnology, Shahjalal University of Science and Technology, Sylhet, Bangladesh; 4 School of Environmental and Life Sciences, The University of Newcastle, Callaghan, NSW Australia; 5 ICAR-Indian Institute of Agricultural Biotechnology, Ranchi, Jharkhand India; 6 Helicobacter Research Laboratory, School of Biomedical Sciences, Marshall Centre for Infectious Disease Research and Training, University of Western Australia, Perth, WA Australia

**Keywords:** Microbiology, Environmental sciences

## Abstract

Carbon sources are considered as critical input for the health and immunity of aquatic animals. The present study investigated the impact of different carbon sources on water quality parameters, carbon to nitrogen (C/N) ratio and microbial community in sediments, and health responses of marron (*Cherax cainii*) under laboratory conditions. Following one week of acclimation, 120 marron were randomly assigned to 12 experimental tanks. There were four treatments including one untreated control and three groups with carbon addition to maintain a C/N ratio of 12 maintained in culture water. Carbon supplementation groups included corn flour (CBC12), molasses (MBC12) and wheat flour (WBC12). At the end of the 60-day trial, MBC12 resulted in the highest sediment C/N ratio, followed by CBC12. Weight gain and specific growth rate were higher in MBC12, compared to control. The protease activity in marron hepatopancreas, total haemocyte count and lysozyme activity in haemolymph were highest in MBC12. Analysis of 16S rRNA sequence data of tank sediments revealed increased bacterial alpha diversity in MBC12 and WBC12. Proteobacteria was the most abundant phylum in MBC12 (88.6%), followed by control (82.4%) and CBC12 (72.8%). *Sphingobium* and *Novosphingobium* were the most abundant genera in control and MBC12 groups, respectively. Higher *Aeromonas* abundance in CBC12 and *Flavobacterium* in WBC12 were observed. Overall results indicated that MBC12 led to improved water quality, retaining high C/N ratio and enriched the bacterial populations in sediments resulting in improved growth and immune performance of marron.

## Introduction

Marron (*Cherax cainii*) is an important farmed freshwater crayfish in Western Australia^1^. It has a significant potential to expand and improve its farming productivity^2^ due to its large size, mobility and omnivore^3^. Numerous studies on marron^4,5^ have been conducted and further research is in progress^6,7^ with the aim to enhance marron productivity through feeding of formulated diets and improving the management practices. Like other decapods, the natural habitat of marron is the sediment–water interface which is the boundary between the bottom and overlying water column. The detritus/sediment is recognised as a vital *in-situ* food source for marron nutrition^8^. However, research efforts to decode the sediment characteristics in marron aquaculture is still in infancy stage, and its effects on marron growth and health parameters are obscure.

In aquaculture systems, a large amount of undigested feed limits the conversion of carbon (C) and nitrogen (N) into final biomass^9^. For instance, a maximum of 16% (C) and 37% (N) were found to be converted into the flesh of freshwater prawn (*Macrobrachium rosenbergii*)^10^, tiger shrimp (*Penaeus monodon*)^11,12^ and white leg shrimp (*Litopenaeus vannamei*)^9^ farmed in earthen ponds. The retained percentage could be even lower to 10% (C) and 6% (N) due to low shrimp survival in integrated rice-shrimp culture systems^13^. A relatively high percentage of unused N settles as sediments in ponds^12^ or in tanks^14^. From the total inputs, up to 53% of the N can be deposited as tank sediments^11,14^. Hence, it is absolutely imperative to optimise feed utilisation in order to reduce the amount of undigested feed and the deposition of N wastes in sediments.

Carbon to nitrogen (C/N) ratio is an integral element that plays a vital role in converting N wastes into bacterial biomass^15–17^ and thereby promoting circular growth of blue economy. Analysis of the C/N ratio in sediment is vital for gaining insight into the source of essential elements such as C and N in the aquaculture pond environment^15,18^. Therefore, managing the C/N ratio and understanding their interaction with microbial communities in sediment is necessary for water quality control and animal performance in aquaculture ecosystems. By adding external carbon sources into aquaculture rearing systems, the C/N ratio can be manipulated for enhanced nitrogen uptake by heterotrophic bacteria leading to decreased ammonium concentration and increased microbial biomass^19^. Carbohydrates such as corn and wheat flour are not only essential ingredients in the diets of decapod species^2,20,21^ but are in vogue as exogenous carbon sources in aquaculture rearing systems. Supplementation of corn starch and wheat flour are reported to enhance water quality, heterotrophic bacterial biomass, growth and survival of various cultured shrimp species such as pink shrimp (*Farfantepenaeus brasiliensis*)^22^, freshwater prawn^23^ and white leg shrimp^14^. Unlike corn and wheat, molasses are not the prevalent ingredient in crustacean diets including marron, but has been widely used in aquaculture of tiger shrimp^24^, white leg shrimp^25^, and freshwater prawn^26–28^ as an external carbon source. Compared to other exogenous carbon sources, molasses have been validated as superior carbon source in removing ammonia and improving the growth of white leg shrimp^29,30^. Therefore, molasses has the indisputable potential for their use as additional carbon sources in marron aquaculture.

Previous studies on red claw (*Cherax quadricarinatus*)^31^ and red swamp (*Procambarus clarkii*)^32–34^ reported beneficial effects of carbohydrate supplementation. For example, molasses supplementation improved water quality and feed utilization efficiency of red claw^31^. Adding external carbon sources (glucose and wheat bran) enhanced the growth performance and proximate composition of red swamp^33^. Additionally, another study found that narrow-clawed crayfish (*Astacus leptodactylus*), as indicated by their normal haemolymph indices, can adapt well to high stocking densities in the carbon supplementation system^35^. However, similar studies are lacking for marron, necessitating further investigation to elucidate the potential advantages of carbon supplementation on the growth and overall health of marron. It is hypothesized that carbohydrate supplementation might play a vital role in stimulating the assimilation of nitrogenous wastes either suspended in the water column or sediments, thereby improving water quality as well as augmenting physiological and immunological performances of marron. The present experiment aims to assess the effect of three carbohydrate sources, corn flour, wheat flour, and molasses, on the water quality characteristics, C/N ratio and microbial communities of the tank sediments in relation to the growth and health responses of marron under laboratory conditions.

## Results

### Water quality

Water temperature did not differ significantly between treatments during the trial. Overall, pH, dissolved oxygen, and nitrogenous compound levels were significantly higher in control than in carbon-added treatments (Table 1). No significant differences between the three carbon-treated groups were observed in all tested water quality parameters, except for a lower pH level in WBC12 than in the other two carbon-added treatments. Phosphate level in MBC12 was significantly lower than in the control and WBC12; however, it did not differ from CBC12.

**Table 1:** Water quality (mean ± standard error) in different treatments over a 60-day trial.

Parameters (n = 3)	Control	CBC12	MBC12	WBC12
Temp. (^°^C)	21.61 ± 0.01^a^	21.55 ± 0.02^a^	21.59 ± 0.05^a^	21.41 ± 0.1^a^
pH	7.74 ± 0.01^a^	7.46 ± 0.00^b^	7.44 ± 0.02^b^	7.37 ± 0.00^c^
DO (mg L^−1^)	8.24 ± 0.01^a^	7.57 ± 0.02^b^	7.57 ± 0.01^b^	7.58 ± 0.04^b^
Nitrate (mg L^−1^)	0.93 ± 0.01^a^	0.76 ± 0.02^b^	0.74 ± 0.03^b^	0.77 ± 0.01^b^
Nitrite (mg L^−1^)	0.10 ± 0.00^a^	0.08 ± 0.00^b^	0.07 ± 0.00^b^	0.07 ± 0.00^b^
Ammonia (mg L^−1^)	0.05 ± 0.00^a^	0.04 ± 0.00^b^	0.04 ± 0.00^b^	0.04 ± 0.00^b^
Phosphate (mg L^−1^)	0.32 ± 0.00^a^	0.3 ± 0.01^ab^	0.28 ± 0.00^b^	0.31 ± 0.01^a^

*Temp.* temperature, *DO* dissolved oxygen.

Within the same row, data having different superscript alphabet (a, b, c) are significantly different (P < 0.05).

### Sediment C/N ratio

**Figure 1:**
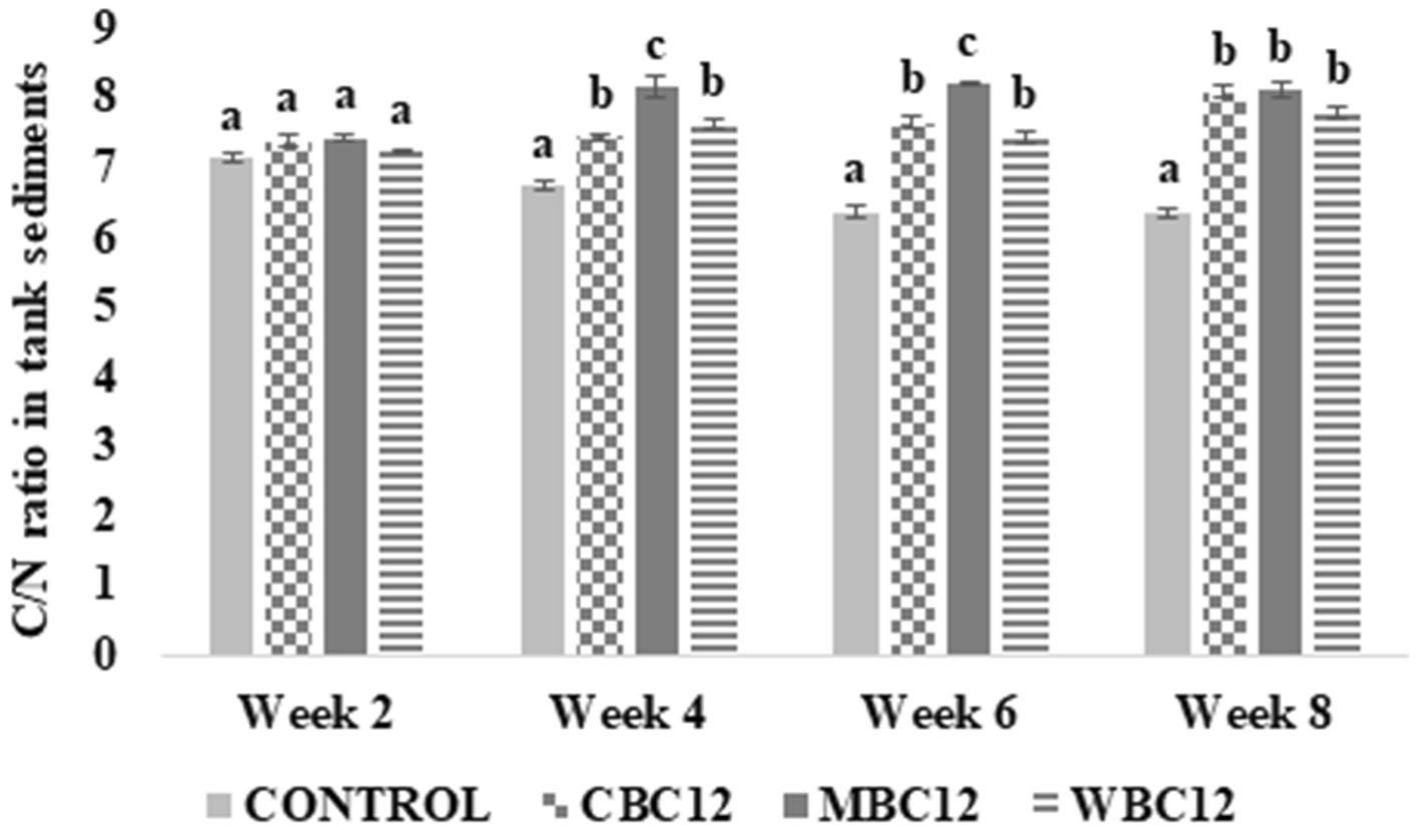
Changes in carbon/nitrogen in the tank sediment in different treatments at four sampling points. The alphabet letters (a, b, c) on the top of each bars indicate statistical difference between treatments (P < 0.05).

Adding different carbon sources in the culture system significantly affected C/N ratio in the tank sediments from week four onwards (Fig. 1). The sediment C/N ratio was significantly higher in MBC12 relative to the control at weeks four, six, and eight of the trial. In the fourth and sixth weeks, there were significant increases in sediment C/N ratio of MBC12. In the eighth week, MBC12 still had the highest sediment C/N ratio (8.12 ± 0.10), followed by CBC12 (8.08 ± 0.08) and WBC12 (7.78 ± 0.08), and the lowest C/N ratio was observed in control (6.36 ± 0.02).

### Bacterial load and composition in tank sediments

#### Sequence statistics and alpha–beta diversities

**Figure 2:**
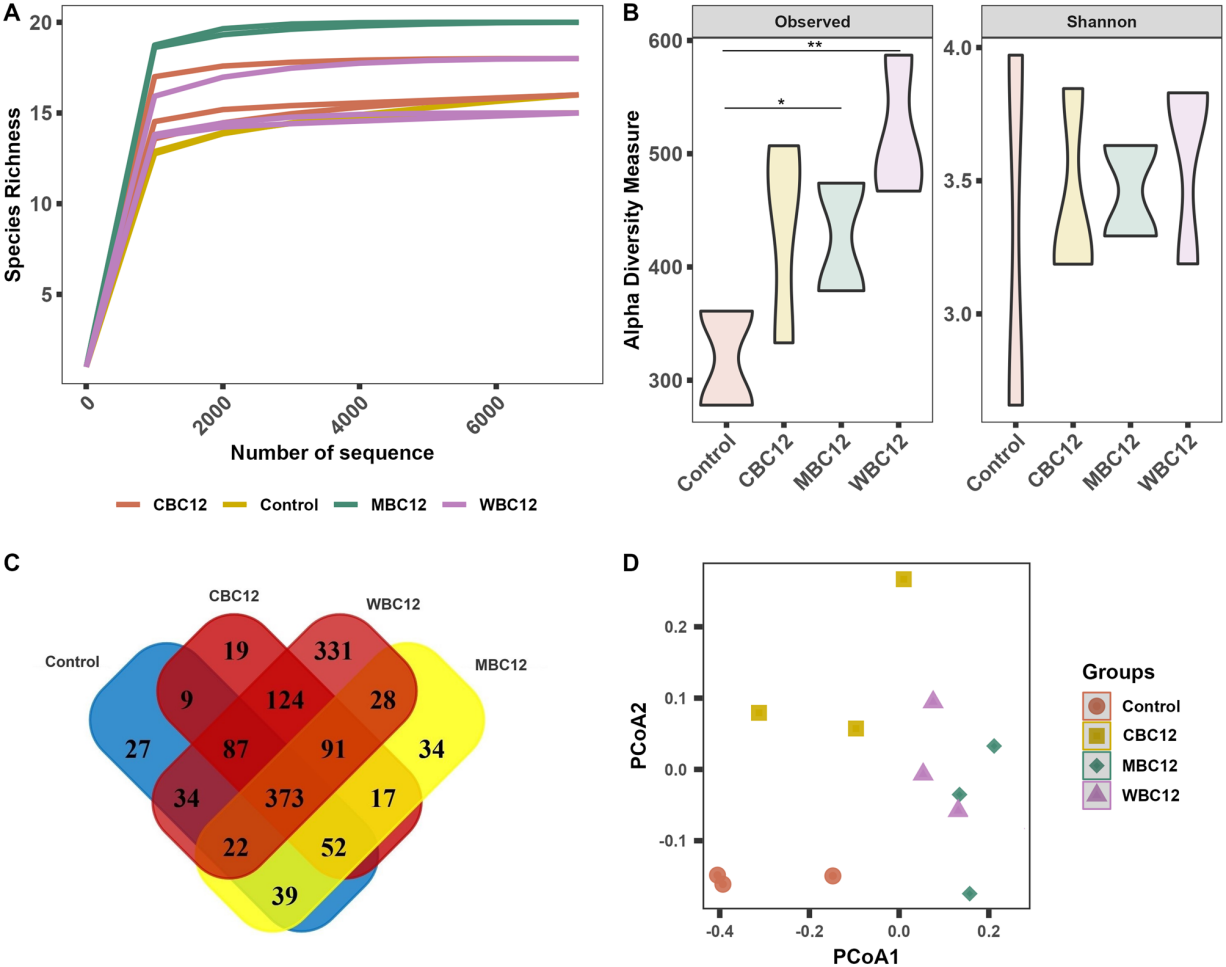
Alpha–beta diversity measurements of bacterial diversity in sediment samples collected from tanks treated with three different carbon sources relative to one control group. (**A**) Rarefaction curve showing the depth and saturation level of 16S rRNA sequence. (**B**) Alpha-diversity measurements in terms of observed species and Shannon index. (**C**) Number of shared and unique ASVs in four different treatments. (**D**) Beta-ordination plot regarding Bray-Curtis dissimilarity of relative abundance

A total of 639,788 quality reads were obtained from 12 samples after trimming, ranging from 47,490 to 127,236 reads. Reads were assigned to 1301 OTUs, 8 phyla, 56 families, and 129 genera. The rarefaction depth curve (Fig. 2A) and Good’s coverage indices (0.992–0.997) indicated that each sample was sequenced at enough depth to capture maximum diversity. The alpha diversity measurements showed that MBC12 and WBC12 had a positive influence on bacterial diversity, compared to the control (Fig. 2B), however WBC12 generated the highest unique OTUs in the sediments (Fig. 2C). The clustering of bacterial OTUs for four different groups was found distinct in the Beta-ordination principal coordinate analysis (PCoA) where PERMANOVA R and P-value of weighted uniFrac metric revealed significantly different of bacterial composition in different groups (Fig. 2D).

#### Microbial composition

**Figure 3:**
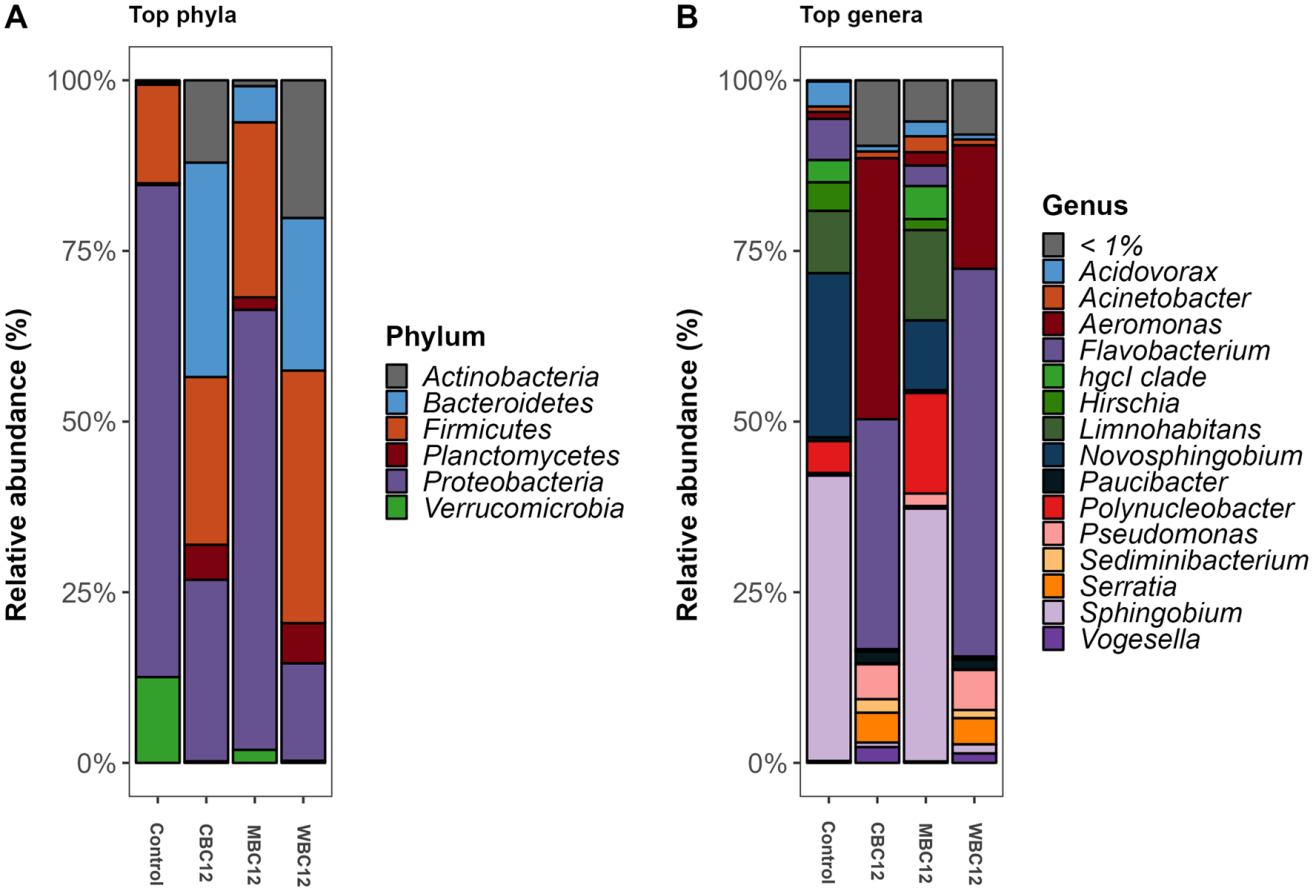
Relative abundance of bacteria at (**A**) phylum and (**B**) genus level. Only OTUs representing at least 1% of the total reads are shown.

At the phylum level, Proteobacteria was the most abundant bacteria in the control (82.4%), CBC12 (72.8%), and MBC12 (88.6%) while Bacteroidetes (56.2%) dominated microbial communities in WBC12 sediments. At the genus level, *Sphingobium* and *Novosphingobium* comprised 62% and 48% of the read abundance in the control and MBC12, respectively, whereas, in CBC12 and WBC12, *Aeromonas* and *Flavobacterium* accounted for 82.2% of the read abundance (Fig. 3A,B).

**Figure 4:**
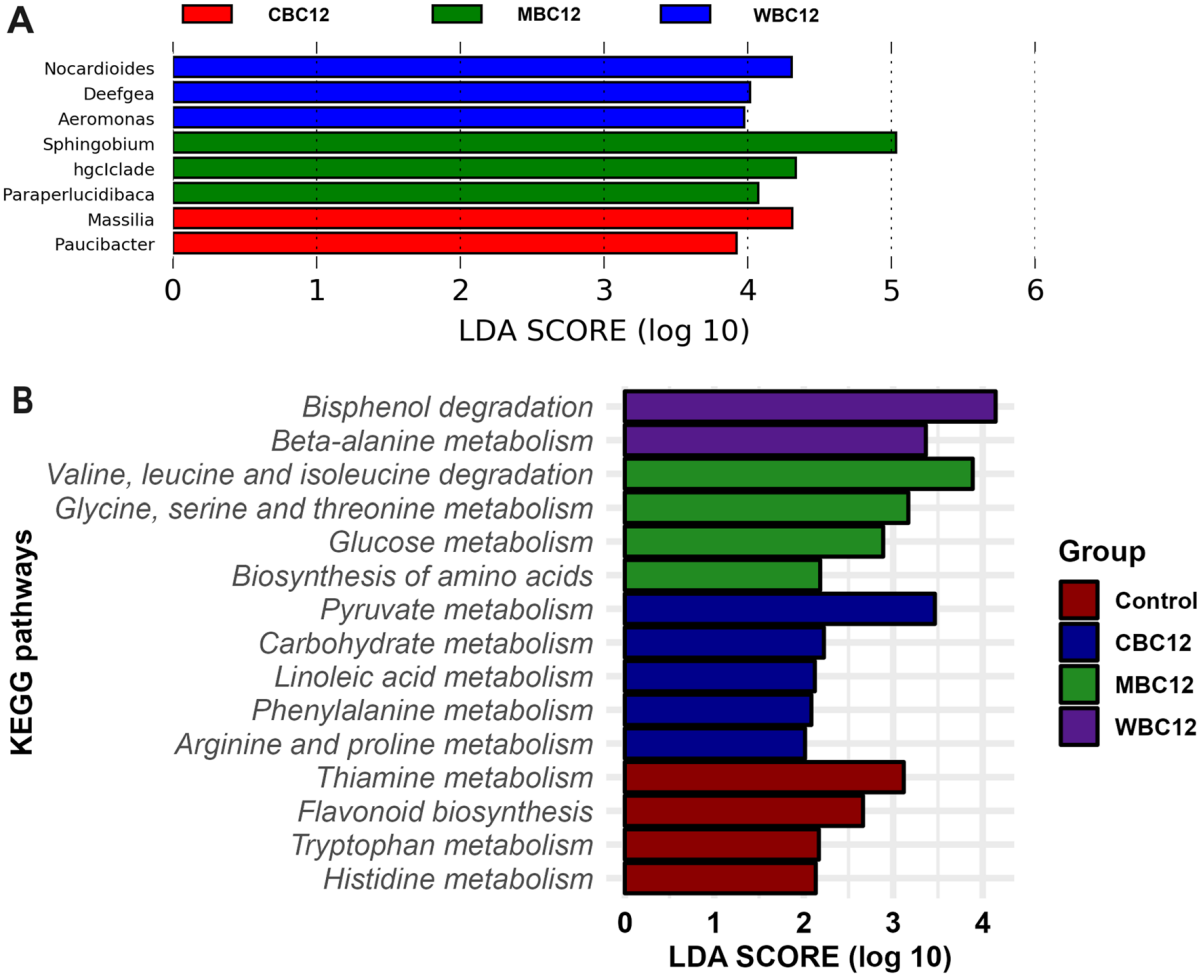
Significantly abundant genera (**A**) and metabolic pathways (**B**) in three different treatment groups with Linear Discriminant Analysis (LDA). No genus had differential abundance in the control group with LDA value 2.0 and 0.05 level of significance.

Alongside these four bacteria groups, *Acinetobacter* in the control and MBC12, *Dechloromonas* in CBC12 and WBC12, *Hirschia* in control, and *Serratia* in CBC12 and WBC12 had more than 1% read abundance in the sediment bacterial communities. Relative to the control group, eight bacteria groups at the genus level had significantly different reared read abundance in three carbon-treated groups. The abundance of *Massilia* and *Paucibacter* was high in CBC12 while MBC12 favoured the growth of *hgcl clade*, *Paraperlucidibaca,* and *Sphingobium* whereas WBC12 promoted the abundance of *Aeromonas*, *Deefgea* and *Nocardioides* (Fig. 4A). PICRUSt2 data showed that CBC12 and MBC12 enriched pathways for carbohydrate, protein and amino acid metabolism. In addition, MBC12 enhanced activities for the biosynthesis and metabolism of amino acids. WBC12 only enriched beta-alanine metabolism compared to others. Control samples on the other hand mostly involved in amino acid metabolism (Fig. 4B).

#### Sediment C/N ratio-taxa correlations

**Figure 5:**
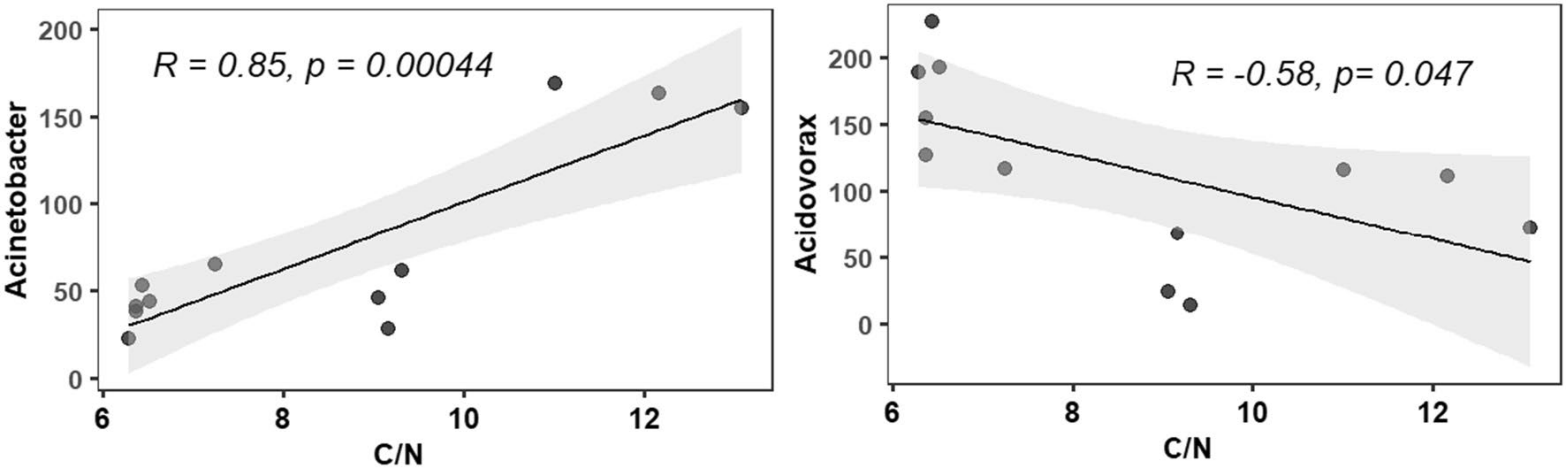
Pearson correlations between the most abundant bacterial taxa and sediment C/N ratio. Significant level at P < 0.05.

Among the correlations between sediment C/N ratio and the relative abundance of most abundant genera, sediment C/N ratio was found to have significant correlations with *Acinetobacter* and *Acidovorax*. C/N ratio was positively and negatively correlated to *Acinetobacter* and *Acidovorax*, respectively (Fig. 5).

### Marron growth performance

The supplementation of different carbon sources into the culture system led to significant changes in WG and SGR of marron; however, it did not significantly affect the survival rate at the end of the trial (Table 2). The highest and lowest growth performances, as measured by WG and SGR were observed in MBC12 and control group, respectively. Similarly, MWI was significantly higher in MBC12 than in the control. No significant differences were observed in moult intervals between treatments. The highest protease activity in the hepatopancreas of marron was also observed in the MBC12 (1.65 ± 0.05 μg/mL), followed by WBC12 (1.34 ± 0.04 μg/mL). In contrast, the lowest protease activity among the treatments was observed in CBC12 (0.99 ± 0.05 μg/mL), which was statistically similar to the control (1.05 ± 0.08 μg/mL).

**Table 2:** Marron growth (mean ± standard error) performance parameters.

Parameters	Control	CBC12	MBC12	WBC12
Initial weight (g)	11.98 ± 0.09^a^	11.5 ± 0.1^a^	11.7 ± 0.2^a^	11.49 ± 0.05^a^
Final weight (g)	14.47 ± 0.06^a^	14.49 ± 0.06^a^	16.15 ± 0.17^b^	15.55 ± 0.5^ab^
Survival (%)	90 ± 0.00^a^	96.67 ± 3.33^a^	93.33 ± 3.33^a^	90 ± 5.77^a^
Weight gain (%)	21.27 ± 1.16^a^	26.38 ± 1.15^ab^	32.23 ± 2.71^b^	28.05 ± 0.58^ab^
SGR (%/day)	0.34 ± 0.02^a^	0.42 ± 0.02^a^	0.5 ± 0.04^b^	0.44 ± 0.01^ab^
MWI (%)	22 ± 1.07^a^	26.53 ± 0.26^bc^	29.36 ± 0.85^c^	23.77 ± 0.5^ab^
MI (days)	32.44 ± 1.28^a^	31.42 ± 1.23^a^	32.67 ± 1.5^a^	31.02 ± 1.65^a^
Protease (μg/mL)	1.05 ± 0.08^a^	0.99 ± 0.05^a^	1.65 ± 0.05^b^	1.34 ± 0.04^c^

*SGR* specific growth rate*, MWI* moult weight increment, *MI* Moult interval.

Rows having different superscript are statistically different (P < 0.05).

### Marron organosomatic indices

**Figure 6:**
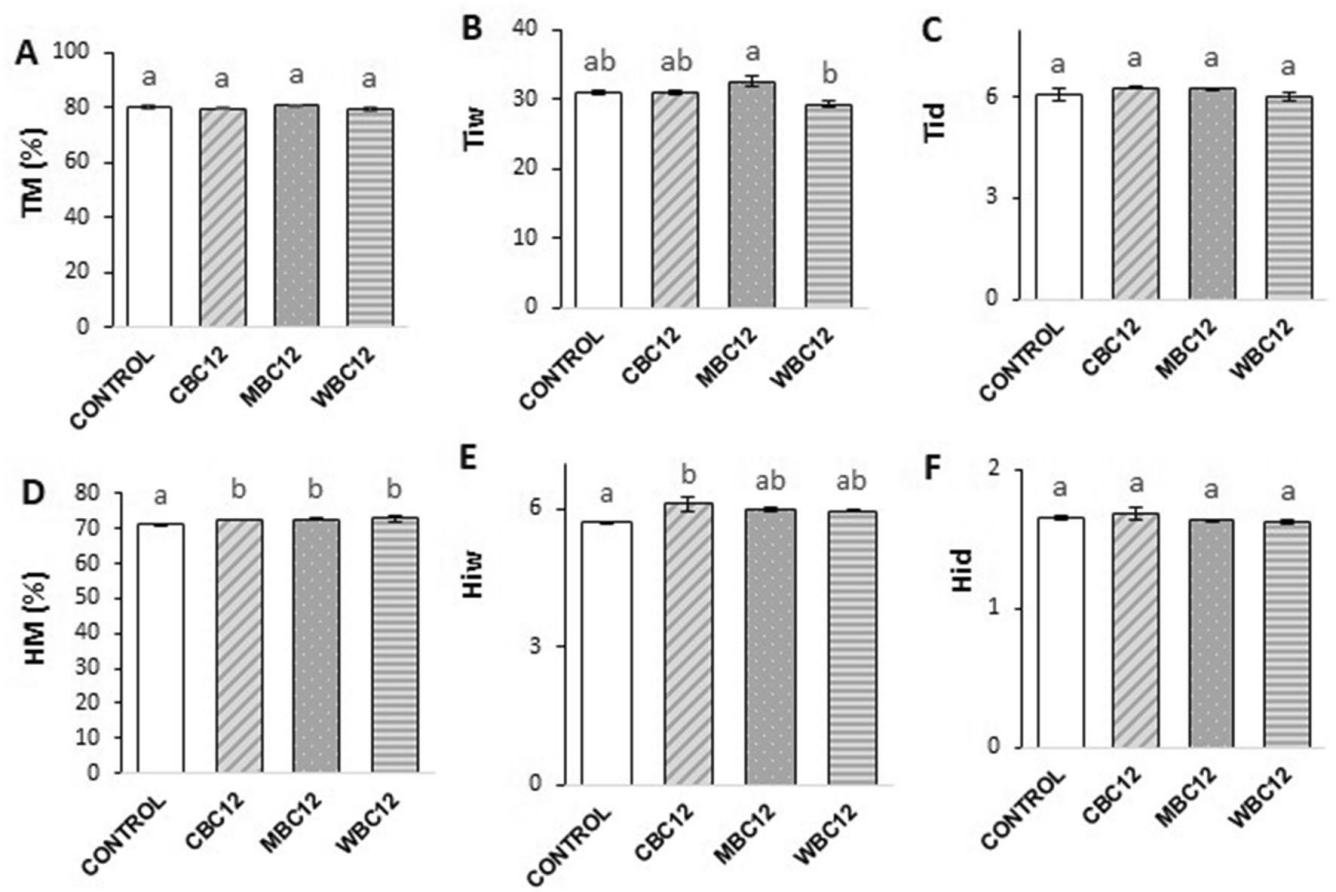
Organosomatic measures (mean ± standard error) of marron treated with different carbon sources. (**A**) Tail muscle moisture content (TM%), (**B**) Dry tail muscle index (Tid), (**C**) Wet tail muscle index (Tiw), (**D**) Hepatopancreas moisture content (HM%), (**E**) Wet hepatosomatic index (Hiw), (**F**) Dry hepatosomatic index (Hid). Different superscript letters (a, b) on the top of the bar represents significant difference at P < 0.05.

Organosomatic indices of marron are presented in Fig. 6. No significant changes in TM (%), Tid, and Hid among the different carbon-treated groups were observed in marron by the end of the experiment. HM (%) in the control was significantly lower than those in all carbon treatments. In MBC12, Tiw was significantly higher than in WBC12; however, there was no significant difference in this parameter between MBC12 and the other two treatments. Hiw was significantly higher in CBC12 than in the control but did not differ statistically from other carbon-treated groups.

### Marron immunological parameters

**Figure 7:**
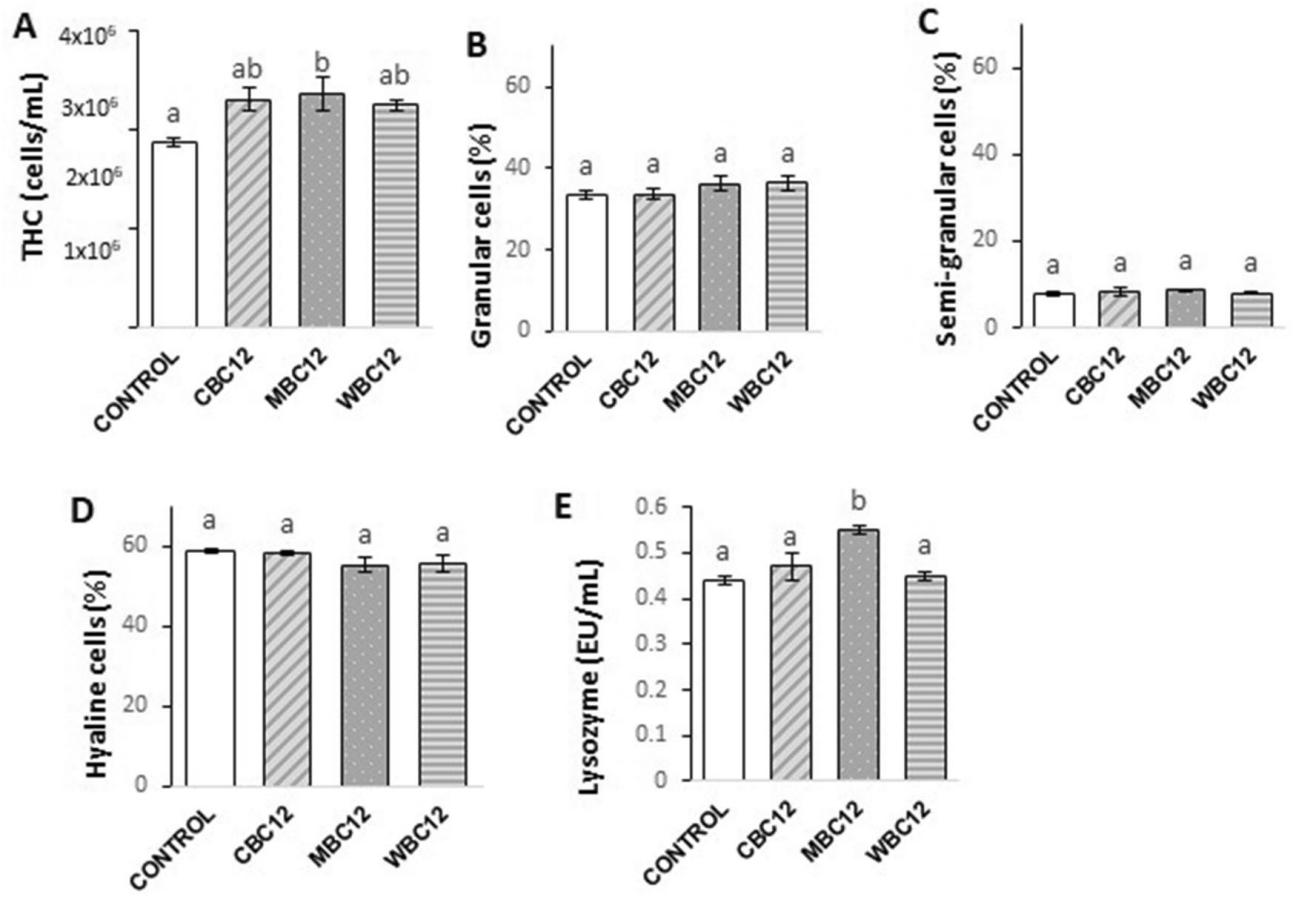
Immune responses of marron in different groups treated with different carbohydrate sources. Different superscript letters (a, b, c) on the top of the bar represents significant difference at P < 0.05. *THC* Total haemocyte count.

Figure 7 shows that by the end of the experiment, THC in the marron haemolymph was significantly higher in MBC12 relative to the control; however, it did not differ from other carbon-treated groups. There was no significant difference in the proportions of granular, semi-granular, and hyaline cells between all groups. However, the highest lysozyme activity was obtained with MBC12, and the differences were significant compared to all other treatments, including the control.

## Discussion

Addition of an external carbon source is absolutely necessary to retain an optimal C/N ratio for promoting the growth of beneficial heterotrophic microorganisms in aquaculture water^15,19^. Moreover, using a suitable external carbon source is vital to enhance the production of the targeted species cultured in this system^36^. The present study demonstrated that adding external carbon sources to marron culture led to beneficial effects on water quality, sediment bacteria, growth performance and the health status of marron. Previous reports^17,37^ demonstrated that water quality could be improved since heterotrophic bacteria can metabolize wastes in carbon-added aquaculture systems. Consistent with studies on other decapod species^14,38^, the present research showed significantly reduction in concentration of nitrogen and phosphorus metabolites following carbon addition in the culture tanks. All carbohydrate sources can affect the immobilization of nitrogenous compounds^39,40^. That could be the reason why various forms of added carbon investigated in the present study were effectively associated with reduced level of toxic nitrogenous wastes. Our results are in agreement with the prior studies on Nile tilapia (*Oreochromis niloticus*)^41^ and white-leg shrimp^42^ that reported lower phosphorus levels in carbon-treated systems. In our study, among the three carbon sources, molasses treatment was found to be more effective in reducing phosphate concentrations in marron culture. It is likely that molasses are made up of simple sugars and thereby readily and rapidly used by microbes^29,43^, which leads to phosphate reduction^28,44^.

However, the inclusion of external carbon into marron culture resulted in declining dissolved oxygen and pH in the water. Similar to our results, low dissolved oxygen and pH values were reported in water of pink shrimp culture when sugarcane molasses and wheat bran were added as external carbon sources^22^. Reduced dissolved oxygen and pH might be correlated with the increased respiration rates of heterotrophic microbial flora that enhanced the carbon dioxide concentration in limited water exchange systems^45^; therefore, applying sodium bicarbonate (NaHCO_3_) is necessary when water pH drops below 7.0^38^.

Sediments in aquaculture systems receive most of the total carbon and nitrogen inputs from exogenous sources^14^. Adding different carbon sources into the culture systems resulted in different amounts of carbon and nitrogen accumulating^14^. In the present study, the sediment C/N ratio declined in control over time while it increased in carbon-added treatments. The lowest C/N ratios were recorded in the control over the entire trial. These results imply that nitrogenous wastes accumulated more in the sediment of the control but have been efficiently decomposed by bacteria in treatments that received carbon supplementation. The highest C/N ratio retained in the sediments of the molasses-treated group could be due to the greater solubility of molasses in the tank water^29^. Molasses produce higher levels of dissolved organic carbon compared to the complex carbon sources of corn and wheat flour, which often require additional time for microbial degradation prior to the carbon source being utilisable by microorganisms^29^. In addition, high C/N ratio could benefit the diversity and community structure of microbial communities in tank sediments^4^.

In the present study, analysis of 16S rRNA sequence data exhibited that adding carbon sources significantly increased bacterial diversity in the sediments. The augmented bacteria in the sediments with wheat flour are reported to play diverse roles, including degradation of complex organic compounds such as atrazine, isoprene by the *Nocardioides*, hydrolytic *Deefgea* with unknown soil function, and one of the most diverse environmental bacteria *Aeromonas* that is mostly pathogenic to the aquatic species^46,47^. On the other hand, increased bacteria abundance with molasses supplementation are primarily involved in the degradation of contaminated soil by *Sphingobium*, organic waste decomposition by *hgcl-clade*^48^, and seawater bacteria *Paraperlucidibaca*^49^. The augmented genera in wheat flour and molasses attributed to better water quality and improved health status of marron.

In this study, PICRUSt2 has been employed for the metagenome prediction from the bacterial 16S dataset. Due to the low resolution of Illumina amplicon data, the phylogenetic classification has been restricted to the genus level only, and therefore, extraction of metabolic information from short-reads is difficult and not precisely accurate. However, PICRUSt2 has a co-efficiency of more than 80 for metagenome prediction for environmental samples^50^. Alongside the building blocks of protein, amino acids are the primary sources of energy for the gut and hepatopancreas of crustaceans^51^. Carbohydrate is essential, but the requirement for aquatic animals, including crustaceans, is very low^52^, yet enrichment of glucose and carbohydrate metabolism by CBC12 and MBC12 is an indication of balanced carbohydrate, protein and amino acids biosynthesis and metabolism. These findings also support our previous studies with marron and different fed supplementations wherein elevated protein and amino acids activity positively linked to better immune and gut health of marron^53,54^. No toxic activity or biofilm formation signifying no negative impacts of carbon supplementation on gut health and metabolism and the possibility of CBC12 and MBC12 to be used in marron aquaculture.

One of the most significant findings of this study is the correlation between bacterial communities and C/N ratio in the sediments. An increased C/N ratio is linked to better water quality features in aquaculture by removing toxic nitrogenous compounds such as ammonia^55^. We found that *Acinetobacter* positively correlated to C/N ratio in the sediments. This is the bacterial group that can increase C/N ratio through the evolution of CO_2_^56^. Hence, an increase in relative abundance for *Acinetobacter* in the molasses supplemented group might be associated with a higher C/N ratio in the sediment. No parallel reports are available on the negative correlation between *Acidovorax* and C/N ratio in the sediment to further substantiate the findings. Therefore, future research should be focussed on microbial community network and sediment C/N ratio to better understand taxa-environmental correlation under marron aquaculture. However, despite having higher bacterial diversity in the sediment of both wheat flour and molasses added groups, the overall data largely suggest beneficial effects of molasses in terms of beneficial bacteria, and C/N ratio retention.

The addition of external carbon sources significantly benefited the growth performance of various fish^57,58^ and shrimp species^14,38^. In the present study, all three exogenous carbon sources included in the marron culture did not significantly affect marron survival, marron weight gain; however specific growth rate, and moult weight increment were improved. The survival rate of the marron in this study was comparable to those reported in a previous study^8^, which may be due to the same approach of housing the marron individually to prevent cannibalism. Moreover, heterotrophic bacteria presented in the carbon-enriched treatments could assimilate nitrogenous waste and convert it into protein-rich microbial biomass, thereby supplying the nutrients for the growth of aquatic organisms^30,43^. Furthermore, decapod growth is restricted by the ability of their digestive systems to break down and absorb specific nutrients^59^. Red swamp crayfish raised in the culture system with external carbon addition obtained higher hepatopancreatic pepsin activities^33^. Results of the current study reveal that molasses treatment increased protease activity in marron hepatopancreas, resulting in the highest moult weight increment and specific growth rate of marron which is in line with the results documented in other studies^29,30^. The lower specific growth rate of marron in the current study is in contrast to the findings of Tulsankar et al.^8^, which might be associated due to a larger size of marron used as experimental animal.

Moisture and immunological parameters have been widely used as robust indicators of marron health conditions^2^. High dry tail muscle and hepatopancreatic indices indicate greater energy storage in marron flesh and hepatopancreas, respectively^2^. In our study, carbon supplementation had significant impacts on some moisture contents of marron such as hepatopancreas moisture, wet hepatosomatic and wet tail muscle indexes. In contrast to our results, hepatopancreas index and meat yield were unchanged in red swamp crayfish cultured in the system with carbon addition relative to the control^33^. The different crayfish species and carbon sources used in these two studies could be responsible for the different outcomes. El-Sayed et al.^36^ reported that some probiotic agents presented in carbon-added systems can augment the immune system in shrimp. In the present study, molasses addition improved the immunological parameters, including THC and lysozyme activity. High THC and lysozyme activity are associated with better marron health conditions^60^. As an external carbon source, molasses has proven its effectiveness in strengthening the immune system of various farmed animals. For example, Zhao et al.^61^ reported significantly higher THC in the haemolymph of the shrimp treated with molasses relative to shrimps reared with other carbon sources. Similar results were confirmed by farming white-leg shrimp with molasses supplementation^29,30^. The findings of similar proportions of differential haemocyte cells in marron reared in carbon-enriched environments are reported for the first time in the scientific literature. These blood cells function in wound healing and phagocytosis; thereby, their fluctuations in the differential haemocyte profile suggest a response to foreign invaders^62,63^. Further research is needed to clarify roles of haemocyte cells in marron health when the animals are cultured in carbon-supplemented systems.

In conclusion, adding carbon sources to the marron culture system led to improved performance of marron which are reflected on the water quality improvement, and high C/N ratio retention in the sediments. Among all the carbon sources tested, the molasses treated group remarkably fostered the diversity of bacterial populations in tank sediments. These conditions further contributed to improved growth and health performances of marron. Future research efforts on the optimisation levels of molasses will provide insightful understanding for better management and profitability of commercial marron farming and for promotion of sustainable blue economy.

## Materials and methods

No animal ethic was required as the study involved invertebrates.

### Preparation of microbial inoculum water for carbon-added treatments

Microbial inoculum water was prepared following the procedure provided by Ahmad et al.^57^ with some modifications. Three indoor plastic tanks were filled with 150 L freshwater. Each tank contained 3000 g of bottom soil from the commercial Blue Ridge Marron farm, Manjimup, Western Australia (34°12′22″ S, 116°01′01″ E), 1.5 g ammonium sulphate (NH)_4_SO_4_, and 60 g selected carbon sources being either corn flour, molasses or wheat flour. These tanks were kept in a laboratory with controlled temperature (22 °C), wherein a photoperiod of 12-h light and 12-h dark was provided with optimum aeration for 24 h to stimulate the growth of heterotrophic microbial biomass.

### Experimental design

A total of 120 marron with an initial body weight of 11.67 ± 0.11 g (mean ± SE) was purchased from Blue Ridge Marron farm. The marron were acclimated in experimental tanks for one week before being weighed at the start of the experiment, which lasted for 60 days. There were one clear water treatment (control) and the supplementation of corn flour, molasses and wheat flour as treatments of carbohydrate sources to maintain a C/N of 12 in the culture water, namely CBC12, MBC12, and WBC12, respectively. A complete randomized design with three replications per treatment was employed. Experimental tanks with 300 L water capacity were filled with 250 L freshwater for the control tanks and 200 L for carbon-treated tanks, and stocked with ten marron per tank housed individually in black plastic mesh cages. Throughout the experiment, a 12-h cycle of light and dark was maintained. Fishmeal based diet was formulated for marron containing 29.93% crude protein following the standard protocol as described in our previous study^54^. Marron were fed once daily in the evening, and the feeding rate was set at 3% of the total stocked biomass during the acclimation and also during the experimental period. Uneaten feed and faeces were removed out from the tanks every morning to prevent the deterioration of water quality.

The prepared inoculum was used to inoculate the experimental tanks once at the beginning of the experiment, 50 L of microbial inoculum water per tank, to provide a known community profile of the microbial source to imitate the microbial community in pond culture. Then to maintain the C/N ratio of 12 in the rearing water, method of Perez-Fuentes et al.^58^ was followed by daily adding the carbon source into the experimental tanks after feeding marron with a formulated feed. The amount of carbohydrate used per treatment was determined by the content of protein (%) in the formulated feed and the amount of feed supplied during the experiment, not considering the amount of carbon contained in the feed. Assuming that protein is 16% nitrogen and that marron excrete 65% of protein as nitrogen. In 1000 g of marron feed (29.93% crude protein), there is 47.89 g nitrogen. Of the nitrogen used, 31.13 g nitrogen is excreted into water by marron. Corn flour, molasses, and wheat flour contained 39.55%, 40.38%, and 39.53% carbon, respectively. Therefore, to maintain a C/N ratio of 12 in the rearing water, 944.37 g corn flour, 924.96 g molasses, and 944.85 g wheat flour are required for 1000 g of feed supplied. Based on this calculation, the amount of corn flour, molasses, and wheat flour required per treatment per day was 9.78 g, 9.74 g, and 9.77 g, respectively. During the experiment, no water exchange was done in all treatments. However, every week, 30% of the water from each culture tank containing sediments was transferred to another empty tank, and the water was then carefully returned to the original tank after the sediments were collected.

### Data collection

#### Water quality

The experiment was carried out in a wet laboratory room at a constant temperature of 22 °C. Air diffusers and air pumps were used to continuously aerate the tanks. Daily measurements were made of water temperature, pH and dissolved oxygen (DO) using digital pH/ºC CyberScan pH 300 and a YSI 55 DO meter, respectively. Other water parameters were measured weekly. According to the manufacturer’s instructions, a HACH colorimeter was used to analyse the concentrations of nitrite (NO2^−^), nitrate (NO3^−^), ammonia (NH4^+^) and phosphate (PO4^−^), using the diazotization method (low range 0–0.35 mg/L), cadmium reduction method (high range 0–30 mg/L), salicylate method (0–0.5 mg/L) and amino acid method (0–30 mg/L), respectively. All chemicals used to analyse these parameters were obtained from ROWE Scientific Pty Ltd.

#### Sampling of sediments

##### Sediment sampling for analysing C/N ratio

On weeks 2, 4, 6 and 8 of the experiment, sediment samples were collected for analysing the C/N ratio. Tank sediments were collected as mentioned above. The sediments along with 30% of the water from culture tanks were siphoned out to empty tanks. When the solid matters settled as sediments, sediment samples were collected and the supernatants were then returned to the original tanks. These sediment samples were oven dried at 60 °C for 24 h.

##### Assessment of carbon/nitrogen ratio in tank sediments

Centrifuged sediment samples were oven-dried at 60 °C for 24 h, then pulverized to pass through a 0.25 mm mesh screen to obtain prepared sediment samples. A PE 2400 CHN Elemental analyser was used to measure carbon and nitrogen percentages in these samples. The combustion tube packing came with the equipment and consisted of silver vanadate, silver tungstate on magnesium oxide and EA-1000 (chromium oxidiser). In the reduction tube, there were Perkin-Elmer Copper Plus ( +) and Cuprox. Approximately 2 mg of prepared sediments were weighed into a tin capsule with a Perkin-Elmer AD-6 Autobalance, and the percentages of carbon and nitrogen were obtained to calculate the C/N ratios^64^.

### Sediment sampling for analysing microbiota

On the final week of the experiment (days 52, 54, and 56), sediment samples were collected as described previously for analysing the microbial community. A pool was produced by thoroughly mixing the sediment samples from three replicated tanks within each treatment on a respective day. After pooling, three sediment samples of each treatment were created. These samples were then centrifuged for 10 min at 10,000 *rpm* to obtain sedimentary pellets for microbial composition analysis (n = 3 per treatment).

### Assessment of microbial composition in tank sediments

#### Illumina sequencing

Bacterial DNA from sediment samples was extracted using DNeasy Power Soil Kit (Qiagen, Hilden, Germany) following the manufacturer’s instructions. DNA quantity was measured using NanoDrop Spectrophotometer (Thermo Fisher Scientific, USA), followed by dilution to 50 ng/μl final concentration. A total of 35 cycles of amplification for 50 µl final reactions were conducted in a BioRad S100 Gradient Thermal Cycler (Bio-Rad Laboratories, Inc., Foster City, California, USA) containing 2 µl template DNA, 1 µl of each V3-V4 sequencing primers (Part # 15,044,223 Rev. B), 25 µl of Hot Start *Taq* 2X Master Mix (New England BioLab Inc., USA) and 21 µl DEPC treated water. AMPure XP beads were used to process and clean amplified PCR products, and then, in accordance with the Illumina standard methodology (Part # 15044223 Rev. B), amplicon barcoding was performed using a secondary PCR. Samples were sequenced up to 50,000 reads on an Illumina MiSeq platform (Illumina Inc., USA) using a v3 kit (600 cycles, Part # MS-102-3003).

#### Bioinformatics and data analysis

The FastQC pipeline was used to check 16S rRNA sequence quality^65^. Low quality (Q < 20) and short reads (l < 200) were trimmed using the Sickle program. Micca otu (v1.7.0) pipeline was utilised for merging the reads, filtering of chimeric sequences, open-reference clustering of sequences into operational taxonomic units (OTUs) at 97% similarity threshold level, and removing singletons OTUs^66^. Phylogenetic information of each representative OTU was extracted at 97% similarity against the SILVA database (v132 release)^67^. PASTA algorithm and FastTree GRT + CAT models were utilised for multiple sequence alignment and phylogenetic tree construction^68^. The QIIME pipeline (version 1.9.1) and R packages were used to calculate the alpha and beta diversities after an even rarefaction depth value of 40,672 was chosen. Utilising ANOSIM (1000 permutations), non-parametric statistical tests of the distance metric were conducted. Beta-ordination (principal coordinate analysis-PCoA) was performed relying on Bray–Curtis dissimilarity of weighted Unifrac-matric in the microbiomeSeq R package^69^. Significant differential abundance at genus level were found using Linear Discriminant Analysis at a 0.05 level of significance^70^. Metagenome prediction of functional features from 16S rRNA dataset was performed using PICRUSt2 pipeline (v2.2.0) which predicts the function of classified bacteria based on marker gene^50^. Correlation between environmental variables (sediment C/N) and sediment bacterial abundance was performed in terms of the “Pearson” correlation coefficient in microbiomeseq and phyloseq R package. One-way analysis of variance (ANOVA) with Tukey’s HSD was used to calculate any significant differences (P < 0.05) between treatment groups in R Studio.

### Calculations

#### Marron growth

Marron were counted and weighed at the start and conclusion of the experiment. They were also weighed after each moult. Survival rate, percentage weight gain, specific growth rate (SGR), moult interval (MI) and moult weight increment (MWI) were determined using the formulae as follows:

$${\text{Survival rate }}\left( \% \right) = \, \left( {\sum {\text{harvested marron}}} \right)/\left( {\sum {\text{stocked marron}}} \right) \times { 1}00$$

$${\text{SGR }}\left( {\% /{\text{day}}} \right) = \left[ {\left( {{\text{ln final weight}} - {\text{ln initial weight}}} \right)/{\text{days}}} \right] \, \times {1}00$$

$${\text{Weight gain }}\left( \% \right) = \left( {{\text{final weight}} - {\text{initial weight}}} \right)/{\text{ initial weight}}$$

$${\text{MI }}\left( {{\text{day}}} \right) = {\text{ the number of days to moult}}$$

$$MWI \left(\%\right) =\frac{\left(weight\,after\,moult-weight\,before\,moult\right)x100}{weight\,before\,moult}$$

### Protease activity

At the conclusion of the experiment, one marron from each tank was collected for hepatopancreatic protease assay. Subsamples of 0.2 g of hepatopancreatic tissue were homogenised on ice in 2 ml phosphate buffer saline. These subsamples were then centrifuged for 10 min at 10,000 rpm. The supernatants were used for enzyme activity analysis. Activities of protease were recorded as the difference in absorbance recorded at 450 nm using diagnostic reagent kits, following the instructions provided in the Thermo Scientific™ Pierce™ Protease Assay Kit.

### Marron organosomatic indices

To measure the marron organosomatic indices, one individual from each tank was dissected at the end of the trial. All hepatopancreatic lobes and the complete mass of muscle tissues from the marron abdomen were weighed and then dried for 24 h in the oven at 105 °C to determine the moisture contents and hepatosomatic indices. Organosomatic indices and moisture content were calculated using the following calculations previously described by Fotedar^2^.

$${\text{Tail muscle moisture }}\left( {{\text{TM }}\% } \right) = \, \left( {{\text{WTwet }}{-}{\text{ WTdry}}} \right) \, \times { 1}00/{\text{WTwet}}$$

$${\text{Dry tail muscle index }}\left( {{\text{Tid}}} \right) = {\text{ WTdry }} \times { 1}00/{\text{Wt}}$$

$${\text{Wet tail muscle index }}\left( {{\text{Tiw}}} \right) = {\text{ WTwet}} \times { 1}00/{\text{Wt}}$$

$${\text{Hepatopancreas moisture }}\left( {{\text{HM }}\% } \right) = \left( {{\text{WHwet }}{-}{\text{ WHdry}}} \right) \, \times { 1}00/{\text{WHwet}}$$

$${\text{Dry hepatosomatic index }}\left( {{\text{Hid}}} \right) = {\text{ WHdry }} \times { 1}00/{\text{Wt}}$$

$${\text{Wet hepatosomatic index }}\left( {{\text{Hiw}}} \right) = {\text{ WHwet }} \times { 1}00/{\text{Wt}}$$

where, WTwet: weight of wet tail muscles (g); WTdry: weight of dry tail muscles (g); Wt: total weight of marron (g); WHwet: weight of wet hepatopancreas (g); and WHdry: weight of dry hepatopancreas (g).

### Marron immunological parameters

For analysis of total haemocyte count (THC), differential haemocyte count (DHC – granular cells, semi-granular cells, and hyaline cells), and lysozyme activity; samples of 0.2 mL haemolymph was collected from marron pericardial cavity with a 1 mL syringe filled with 0.2 mL anticoagulant solution. Marron THC and DHC were assessed using methods described previously^71^. THC was counted freshly in both grids of a haemocytometer (Neaubauer, Germany) while haemolymph samples were stained with May-Grunwald and Giemsa on glass slides before counting DHC under a microscope. The percentages of three different haemocyte types per 200 cells on each slide were determined.

$${\text{THC }}\left( {{\text{cells}}/{\text{mL}}} \right) = \, \left( {{\text{cells counted }} \times {\text{ dilution factor }} \times { 1}000} \right)/{\text{grid volume}}$$

$${\text{Granular cell }}\left( \% \right) = \, \left( {{\text{number of granular cells}}/{2}00} \right) \times {1}00$$

$${\text{Semi}} - {\text{granular cell }}\left( \% \right) = \, \left( {{\text{number of semi}} - {\text{granular cells}}/{2}00} \right) \times {1}00$$

$${\text{Hyaline cell }}\left( \% \right) = \, \left( {{\text{number of hyaline cells}}/{2}00} \right) \times {1}00$$

Lysozyme activity in marron haemolymph was performed in a 96-well microplate (Iwaki, Japan) using the turbidimetric method as described previously^72^ with some modifications. Each well contained 100 µL haemolymph sample and 100 μL *Micrococcus lysodeiktikus* suspended in 0.25 mg/mL PBS (Sigma-Aldrich, USA) with two wells for each sample. During 16 min, the absorbance at 450 nm was measured every two minutes. Lysozyme activity was determined as the amount of enzyme causing in a decline in absorbance of 0.001/min. Lysozyme activities were presented as units/mL of haemolymph (EU/mL).

## Data Availability

Paired-end raw fastq amplicon sequences have been deposited to the National Centre for Biotechnology Information (NCBI) under the Bioproject accession number PRJNA1015270. All other data are available upon request to the corresponding authors.
